# Investigating the Dialysis Treatment Using Hollow Fiber Membrane: A New Approach by CFD

**DOI:** 10.3390/membranes12070710

**Published:** 2022-07-15

**Authors:** Hortência L. F. Magalhães, Ricardo S. Gomez, Boniek E. Leite, Jéssica B. S. Nascimento, Mirenia K. T. Brito, Morgana V. Araújo, Daniel C. M. Cavalcante, Elisiane S. Lima, Antonio G. B. Lima, Severino R. Farias Neto

**Affiliations:** 1 Department of Chemical Engineering, Federal University of Campina Grande, Campina Grande 58429-900, Brazil; hortencia.luma@gmail.com (H.L.F.M.); severino.rodrigues@ufcg.edu.br (S.R.F.N.); 2 Department of Mechanical Engineering, Federal University of Campina Grande, Campina Grande 58429-900, Brazil; antonio.gilson@ufcg.edu.br; 3 Department of Process Engineering, Federal University of Campina Grande, Campina Grande 58429-900, Brazil; boniek3@gmail.com (B.E.L.); jbsnascimento@uesc.br (J.B.S.N.); mireniakalina@gmail.com (M.K.T.B.); morganamva@gmail.com (M.V.A.); limaelisianelima@hotmail.com (E.S.L.); 4 Department of Exact Sciences and Technology, Santa Cruz State University, Ilhéus 46662-900, Brazil; 5 Federal Institute of Education, Science and Technology of Sertão Pernambucano, Serra Talhada 56915-899, Brazil; daniel.cesar@ifsertao-pe.edu.br

**Keywords:** computational fluid dynamics, hemodialysis, membrane

## Abstract

Due to the increase in the number of people affected by chronic renal failure, the demand for hemodialysis treatment has increased considerably over the years. In this sense, theoretical and experimental studies to improve the equipment (hemodialyzer) are extremely important, due to their potential impact on the patient’s life quality undergoing treatment. To contribute to this research line, this work aims to study the fluid behavior inside a hollow fiber dialyzer using computational fluid dynamics. In that new approach, the blood is considered as multiphase fluid and the membrane as an extra flow resistance in the porous region (momentum sink). The numerical study of the hemodialysis process was based on the development of a mathematical model that allowed analyzing the performance of the system using Ansys^®^ Fluent software. The predicted results were compared with results reported in the literature and a good concordance was obtained. The simulation results showed that the proposed model can predict the fluid behavior inside the hollow fiber membrane adequately. In addition, it was found that the clearance decreases with increasing radial viscous resistance, with greater permeations in the vicinity of the lumen inlet region, as well as the emergence of the retrofiltration phenomenon, characteristic of this type of process. Herein, velocity, pressure, and volumetric fraction fields are presented and analyzed.

## 1. Introduction

In Brazil, according to the Brazilian nephrology census carried out in July 2016, approximately 122,825 people are on dialysis treatment due to renal insufficiency. Unevenly distributed throughout the country, there are 747 active dialysis units (4% in the north region, 18% in the northeast region, 7% in the midwest, 49% in the southeast, and 22% located in the south region), responsible for providing this treatment for the population [1].

Renal insufficiency can be defined as the loss of the kidney’s ability to maintain the electrolyte balance of the body and remove the metabolic breakdown products. In its acute phase, the disease causes a rapid reduction in renal function, being reversible if treated properly. However, chronic renal insufficiency, unlike acute, is characterized by the gradual and irreversible loss of these functions [2].

The progressive accumulation of compounds through metabolism, normally excreted by healthy kidneys, is usually known as the uremic syndrome. This symptom of renal insufficiency can lead to a reduction in the filtration rate of the kidneys to values below 5 mL/min [3,4]. The incidence of this disease has gradually increased in recent years, as a reflection of the population aging that needs to live each day with several comorbidities [5].

Patients suffering from renal insufficiency can be treated initially through blood pressure control, medications, and dietary treatments. However, over time, dialysis treatment or kidney transplantation is necessary [2,6]. In hemodialysis therapy, a device known as a dialyzer is used to supply the kidneys’ function. This equipment consists of a hull (shell) fitted with a bundle of tubes (hollow fiber membranes). The dialyzer allows blood to flow through the tubes, enabling the removal of metabolic waste by mass transfer (diffusion and convection) that occurs through a porous membrane between the blood and the dialysis solution (dialysate), contained on the shell side [7].

Clark et al. [8] mention that the dialyzer is equipment consisting basically of three components: the dialysate fluid compartment, the blood compartment, and the membrane. The authors emphasize that the knowledge about the physicochemical phenomena and mechanisms involved in this process contribute to optimizing the operational parameters involved in hemodialysis treatment.

Ding et al. [9], when studying an artificial kidney, observed that the simulation of a dialyzer based on the finite-element method is a viable alternative to investigate the behavior of the concentration and velocity fields inside the hollow fiber membrane, enabling the obtainment of expressive results, useful for predicting the removal rate of toxins from the blood.

Some works have been reported in the literature evaluating the phenomenon of mass transfer in dialyzers (hollow fiber membranes). Among the various experimental works, we can mention Klein et al. [10] and Liao et al. [11]. Among the theoretical works, we can mention Lu and Lu [12], which proposed to analyze the mass transfer in a dialysis system with the concurrent flow in parallel plates. Among the numerical works, we can mention Pstras et al. [13], who presented the main mathematical models used to study/optimize hemodialysis therapy, and Cancillaet al. [14], who developed a CFD model to simulate the hemodialysis process in hollow fiber membrane modules.

In addition to the aforementioned research, numerical works can be found discussing dialysis treatment from the perspective of chemical species transfer, such as Gostoli and Gatta [15], who studied mass transfer in countercurrent and concurrent flow in a capillary; Ding et al. [16], when developing a double porous zone model for mass transfer in a hemodialyzer, and Kanchan and Maniyeri [17], Liao et al. [18], Lu and Junfeng Lu, [6] and Donato et al. [19] when studying the solute transport in hemodialysis membranes with a two-dimensional approach. However, to investigate the dialysis process from the perspective of momentum transport, Choi et al. [20] and Kim et al. [21] modeled the blood as a multiphase fluid.

Hemodialysis is the main treatment for patients with chronic renal insufficiency. The procedure is performed in specialized nephrology services and has an average duration of three to four hours, requiring three weekly dialysis sessions, which makes the treatment very difficult for human beings. Therefore, contributions in this area represent an expectation to improve the quality of life of patients with this comorbidity. Although works are being developed in this area, investigations about fluid dynamics inside the hollow fiber are still scarce, especially dealing with blood flow through a multiphase approach. Therefore, in addition to the aforementioned research, this work aims to investigate the hemodialysis process in a hollow fiber membrane via computational fluid dynamics.

Hemodialysis is the main treatment for patients with chronic renal insufficiency. The procedure is performed in specialized nephrology services and has an average duration of three to four hours, requiring three weekly dialysis sessions, which makes the treatment very difficult for human beings. Therefore, contributions in this area represent an expectation to improve the quality of life of patients with this comorbidity. Although works are being developed in this area, investigations about fluid dynamics inside the hollow fiber are still scarce, especially dealing with blood flow through a multiphase approach, where the blood is modeled as two distinct phases (blood and contaminant). Therefore, in addition to the aforementioned research, this work aims to investigate the hemodialysis process in a hollow fiber membrane via computational fluid dynamics. The innovative aspect in this research deals with each phase’s mathematical consideration. Herein, each involved phase was defined as an identifiable class of material that has a particular inertial response and interaction with the flow field in which it is immersed. Besides, the porous medium is considered as an extra resistance to flow in the porous region in the form of a moment sink by adding a source term to the momentum equation applied in each element in that region. Clearly, it is an attempt to predict the hemodialysis process based in a new mathematical procedures and tools. Thus, the authors strongly recommend that more sophisticated approaches must be investigated from this research.

## 2. Methodology

### 2.1. Problem Description

**Figure 1:**
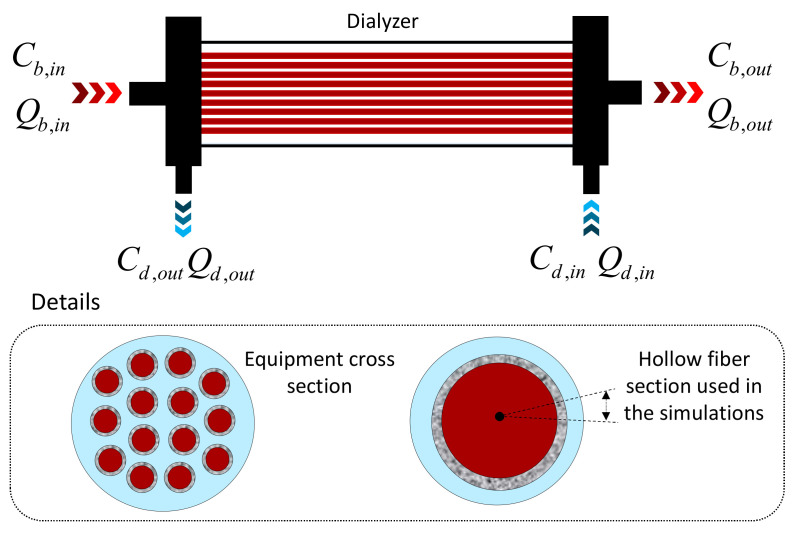
Scheme of the dialyzer operation.

The research is based on a model CT190G dialyzer (Baxter Healthcare Co., McGaw Park, IL, USA) consisting of a hull and a bundle of 12,000 triacetate cellulose hollow fiber. A schematic of the equipment and details of the membrane section used in the methodology can be seen in Figure 1, as described by Liao et al. [18].

**Figure 2:**
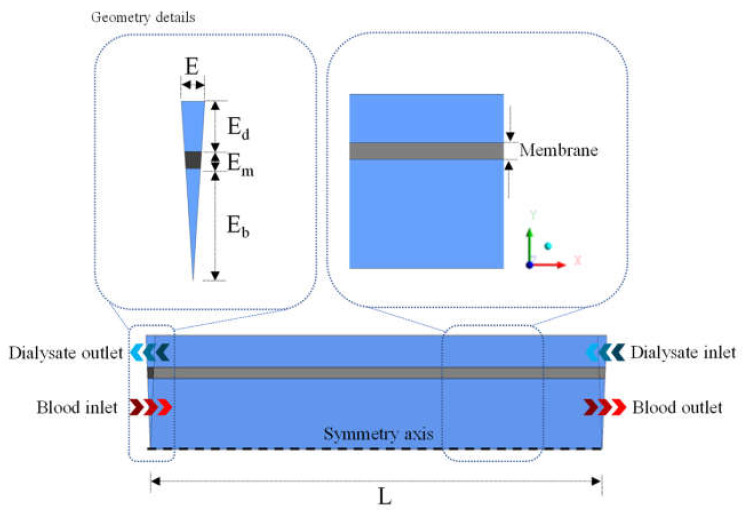
Geometric representation of the hollow fiber membrane.

The hollow fiber membrane consists of three parts: the shell (dialysate flow region), the porous membrane, and the lumen (blood flow region). Inside the equipment, blood (water and urea contaminant) flows into the lumen domain and the dialysate into the shell domain, with the flow in opposite directions (countercurrent), as illustrated in Figure 2. The dimensions of the equipment are listed in Table 1.

### 2.2. Computational Domain

**Figure 3:**
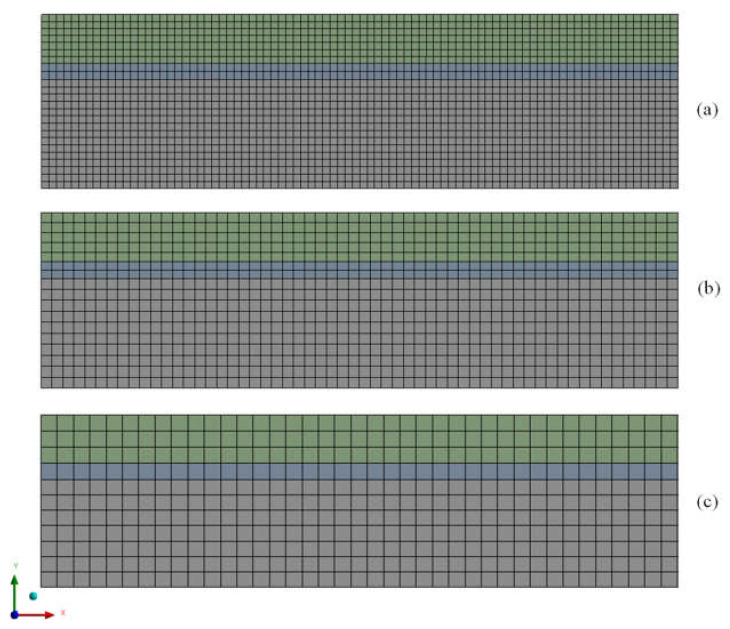
Two-dimensional mesh of the tubular membrane cross-section: (**a**) Mesh $M_{1}$, (**b**) Mesh $M_{2}$, and (**c**) Mesh $M_{3}$.

To perform the numerical simulations, three numerical meshes (*M_1_*, *M_2_*, and *M_3_*) were built using the Ansys^®^ Designer Modeler and Meshing 15.0 software (Canonsburg, PA, USA), as illustrated in Figure 3.

### 2.3. Mathematical Modeling

For the study of the hemodialysis process using a hollow fiber membrane section, the following considerations were assumed:

- Newtonian fluids;
- Flow in a laminar, incompressible, isothermal, and transient regime;
- Constant thermophysical and chemical properties;
- Anisotropic porous medium;
- Negligible gravitational effect;
- The proteins present in the blood were disregarded;
- Adsorption of urea on the membrane contact surface, blockage of membrane pores, formation of concentration polarization layer, and chemical reactions are disregarded;
- Only one section of the hollow fiber membrane is considered, due to the angular symmetry presented by the geometry;
- The Eulerian–Eulerian approach was adopted for multiphase flow.

After due consideration, the mass conservation equation for phase q (Equation (1)) and linear momentum (Equation (2)) can be written as:

- Mass conservation equation for the non-porous media

(1) $$\frac{\partial }{\partial t}\left(f_{q}\rho_{q}\right)+\nabla \cdot \left(f_{q}\rho_{q}{\vec{v}}_{q}\right)=0$$

where f is the volume fraction of the phase q, ${\vec{\nu }}_{q}$ is the velocity vector of the phase q, and ρ is the phase density.

- Linear momentum equation

(2) $$\frac{\partial }{\partial t}\left(f_{q}\rho_{q}{\vec{v}}_{q}\right)+\nabla \cdot \left(f_{q}\rho_{q}{\vec{v}}_{q}{\vec{v}}_{q}\right)=-f_{q}\nabla P+\nabla \cdot {\overset{¯}{\tau }}_{q}+\overset{n}{\underset{p=1}{\sum }}\left({\vec{R}}_{pq}\right)+S_{i}$$

where P is the pressure shared by all phases and ${\overset{¯}{\tau }}_{q}$ is the stress–strain tensor.

The stress–strain tensor is defined by Equation (3) as follows:

(3) $${\overset{¯}{\tau }}_{q}=f_{q}\mu_{q}\left(\nabla {\vec{v}}_{q}+\nabla {\vec{v}}_{q}^{T}\right)+f\left(\lambda_{q}-\frac{2}{3}\mu_{q}\right)\nabla \cdot {\vec{v}}_{q}\overset{¯}{I}$$

where $\mu_{q}$ and $\lambda_{q}$ are the viscosity and shear of phase q.

The term referring to the interface forces, ${\vec{R}}_{pq}$, depends on pressure, friction, cohesion, and other acting effects, and is subject to the conditions of Equations (4) and (5).

(4) $${\vec{R}}_{pq}=-{\vec{R}}_{qp}$$

(5) $${\vec{R}}_{qq}=0$$

Ansys Fluent^®^ software uses an interaction term between the forces, described by:

(6) $$\overset{n}{\underset{p=1}{\sum }}{\vec{R}}_{pq}=\overset{n}{\underset{p=1}{\sum }}K_{pq}\left({\vec{v}}_{p}-{\vec{v}}_{q}\right)$$

(7) $$K_{pq}=K_{qp}$$

where $K_{pq}$ is the interface moment exchange term, given by:

(8) $$K_{pq}=\frac{f_{q}\times f_{p}\times \rho_{p}\times f^{a}}{\tau_{p}}$$

where $f^{a}$ is the drag function, and $\tau_{p}$ is the particulate relaxation time, defined as:

(9) $$f^{a}=\frac{C_{D}\times Re}{18\mu_{q}}$$

(10) $$\tau_{p}=\frac{\rho_{p}\times d_{p}^{2}}{18\mu_{q}}$$

where $C_{D}$ is the drag coefficient, and *Re* is the relative Reynolds number, defined for the primary phase (q) and for the second phase (p), and $d_{p}$ is the diameter of the droplet or bubble of the secondary phase.

The drag coefficient and the relative Reynolds number are calculated using Equations (11) and (12) [22].

(11) $$C_{D}=\left\{\begin{array}{c} 24\times \left(1+0.15\times Re^{0.687}\right)/Re\;\;Re\leq 1000\\ 0.44\;\;\;\;\;\;\;\;\;\;\;Re>1000 \end{array}\right.$$

(12) $$Re=\frac{\rho_{q}\left|{\vec{v}}_{p}-{\vec{v}}_{q}d_{p}\right|}{\mu_{q}}$$

- Linear momentum equation for the porous medium

The porous media model is formed by incorporating extra flow resistance in the porous region, in the form of a momentum sink. This occurs by adding a source term in the momentum equation, applied to the elements of that region. The added source term is composed of two parts: a viscous loss term (Darcy) and an inertial loss term, as seen in Equation (6).

(13) $$S_{i}=-\left(\overset{3}{\underset{j=1}{\sum }}D_{ij}\times \mu \times v_{j}+\overset{3}{\underset{j=1}{\sum }}C_{ij}\frac{1}{2}\rho \times \left|v\right|\times v_{j}\right)$$

where $S_{i}$ is the source term for the *i* (*x*, *y*, or *z*) momentum equations, $\left|\nu \right|$ is the magnitude of the velocity, and μ is the viscosity. The source term added (momentum sink) contributes to the pressure gradient in the porous cell, creating a pressure drop that is proportional to the fluid velocity.

For simple homogeneous porous media, Equation (13) can be rewritten as:

(14) $$S_{i}=-\left(\frac{\mu }{\alpha }\times v_{i}+C_{2}\frac{1}{2}\left|v\right|\times v_{i}\right)$$

where α is the permeability and $C_{2}$ is the inertial resistance factor.

When using the porous medium model, it is considered that the porous cells are completely open, and the only resistance imposed to the flow is given in the form of the viscous (1/α) and inertial ($C_{2}$) resistance coefficients.

For laminar flow through porous media, the constant $C_{2}$ can be considered zero, as the pressure drop is normally proportional to velocity. By disregarding diffusion and convective acceleration, the porous medium model can be expressed by Darcy’s Law, as follows:

(15) $$\nabla P=-\frac{\mu }{\alpha }\vec{v}$$

Therefore, the pressure gradient inside the porous region can be given by:

(16) $$\nabla P_{x}=\overset{3}{\underset{j=1}{\sum }}\frac{\mu }{\alpha_{xj}}{\vec{v}}_{j}\Delta n_{x}$$

(17) $$\nabla P_{y}=\overset{3}{\underset{j=1}{\sum }}\frac{\mu }{\alpha_{yj}}{\vec{v}}_{j}\Delta n_{y}$$

(18) $$\nabla P_{z}=\overset{3}{\underset{j=1}{\sum }}\frac{\mu }{\alpha_{zj}}{\vec{v}}_{j}\Delta n_{z}$$

where $\Delta n_{x}$, $\Delta n_{y}$, and $\Delta n_{z}$ are the thicknesses of the porous medium in the *x*, *y*, and *z* directions, respectively.

#### Conditions Used in Simulations

(a) Initial and boundary conditions

**Figure 4:**
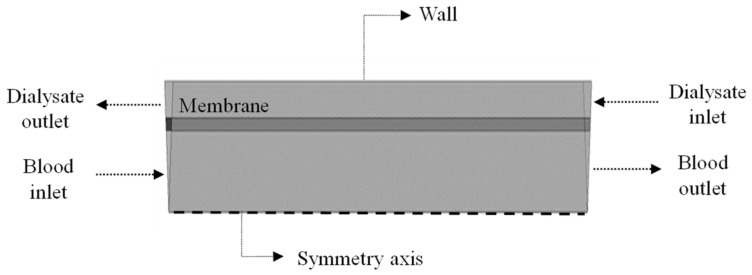
Domain boundaries under study.

To complete the mathematical modeling, the following initial and boundary conditions illustrated in Figure 4 were used:

- Initial conditions

The initial concentration of the contaminant (urea) at the entrance of the lumen, $C_{in}$, at t = 0 s, so that:

(19) $$C=C_{in}$$

where $C_{in}=0.7\;\mathrm{kg}/m^{3}$.

In addition, the fiber was considered to be completely filled with saline solution (water) at time t_0_, before starting the process.

- Boundary conditions

On the axis of symmetry

The following conditions were admitted on the axis of symmetry:

(20) $$\frac{\partial v_{x}}{\partial y}=\frac{\partial v_{y}}{\partial y}=0$$

(21) $$\frac{\partial C}{\partial y}=0$$

(22) $$v_{y}=0$$

In the domain inlets

A condition of constant values for blood and dialysate volumetric fluxes at the domain inlets, in countercurrent, was assumed.

(23) $$Q_{B}=\frac{Q_{Bin}\times \rho_{b}}{N}$$

(24) $$Q_{D}=\frac{Q_{Din}\times \rho_{d}}{N}$$

where $Q_{Bin}$ is the blood volumetric flowrate (water + urea), $Q_{Din}$ is the dialysate volumetric flowrate, and N is the number of dialyzer tubes proposed by Liao et al. [18].

In the domain outlets

A zero-pressure condition at the mass flow outlets was assumed.

(b) Thermophysical parameters of membrane and fluids

The thermo-physical parameters of the fluids and membrane are shown in Table 2.

### 2.4. Studied Cases

To evaluate the influence of the mesh on the simulation results, the grid convergence index (GCI) method was applied to three meshes with different element densities, as shown in Table 3. In the simulations performed, the lumen feed flux, $Q_{Bin}$, the shell feed flux, $Q_{Din}$, the axial viscous resistance, $1/\alpha_{x}$, the radial viscous resistance, $1/\alpha_{y}$, and the urea concentration in the lumen feed, $C_{in}$, were kept constant (Table 4).

After the mesh study was completed, the optimized mesh was selected. With the appropriate mesh, different simulations were carried out, varying the radial viscous resistance. All cases studied are shown in Table 5.

### 2.5. Procedures Used

(a) Mesh evaluation

Celik et al. [23] developed the grid convergence index (GCI), based on the Richardson Extrapolation, for mesh convergence analysis. This method estimates the solution by extrapolating the solutions from existing meshes and by the relative grid convergence index of the meshes produced [24]. Celik et al. [23] report that the procedure for determining the GCI starts by determining the representative mesh size, *h* (Equation (25)), as follows:

(25) $$h={\left[\frac{1}{N_{m}}\overset{N_{m}}{\underset{i=1}{\sum }}\left(\Delta V_{i}\right)\right]}^{1/3}$$

where $N_{m}$ is the number of mesh elements and $\Delta V_{i}$ is the volume occupied by element i.

Using the value obtained in Equation (25), as a reference, meshes are generated, with different numbers of elements. The method determines that the ratio, $r=h/h_{refined}$, must be greater than 1.3 for each generated mesh.

In this methodology $h_{1}>h_{2}>h_{3}$, that is, $h_{1}$ will correspond to the most refined mesh and $h_{3}$ to the less refined mesh, and $\phi_{1}$, $\phi_{2}$, and $\phi_{3}$ will be the respective results of a given variable analyzed. Therefore, the ratios between meshes $r_{21}$ and $r_{32}$ were defined, according to the following equations:

(26) $$r_{21}=\frac{h_{2}}{h_{1}}$$

(27) $$r_{32}=\frac{h_{3}}{h_{2}}$$

Using Equations (28)–(30), the order of convergence, or apparent order (p), is calculated as follows:

(28) $$p=\frac{1}{ln\left(r_{21}\right)}\left|ln\left|\frac{\varepsilon_{32}}{\varepsilon_{21}}\right|+q\left(p\right)\right|$$

(29) $$q\left(p\right)=ln\left(\frac{r_{21}^{p}-s}{r_{32}^{p}-s}\right)$$

(30) $$s=sign\left(\frac{\varepsilon_{32}}{\varepsilon_{21}}\right)$$

where,

(31) $$\varepsilon_{21}=\phi_{2}-\phi_{1}$$

(32) $$\varepsilon_{32}=\phi_{3}-\phi_{2}$$

From Equation (29), it is observed that $q\left(p\right)$ = 0 for $r_{21}=r_{21}$.

According to Paudel and Saenger [25], the value of the constant c that determines the convergence (Equation (33)) is given by:

(33) $$c=\frac{\phi_{1}-\phi_{2}}{\phi_{2}-\phi_{3}}$$

For c>1 there is a monotonic divergence of the solution, 0<c<1 a monotonic convergence, −1<c<0 an oscillatory convergence, and c<−1 indicates an oscillatory divergence.

The extrapolated solutions and the approximate relative errors can be determined by Equations (34)–(36), respectively.

(34) $$\phi_{ext}^{21}=\left(\frac{r_{21}^{p}\times \phi_{1}-\phi_{2}}{r_{21}^{p}-1}\right)$$

(35) $$e_{a}^{21}=\left(\frac{\phi_{1}-\phi_{2}}{\phi_{1}}\right)$$

(36) $$e_{a}^{32}=\left(\frac{\phi_{2}-\phi_{3}}{\phi_{2}}\right)$$

Therefore, based on the equations presented, the Grid Convergence Index (GCI) can be obtained using Equations (37) and (38), as follows:

(37) $$GCI_{21}=\frac{1.25\times e_{a}^{21}}{r_{21}^{p}-1}$$

(38) $$GCI_{32}=\frac{1.25\times e_{a}^{32}}{r_{32}^{p}-1}$$

Equations (25)–(38) were implemented in a VBA/Excel code to perform the calculations, using the ratio, $r_{21}=r_{32}=1.5$.

(b) Validation of the mathematical model

To obtain the radial viscous resistance (in the y-direction) corresponding to the membrane resistance used by Liao et al. [18] in their experiments, six simulations (Cases 04, 05, 06, 07, 08, and 09) were carried out, keeping constant values for feed fluxes and axial viscous resistance $\left(1/\alpha_{x}\right)$, and varying the radial viscous resistance $\left(1/\alpha_{y}\right)$.

After the end of each simulation, a process parameter known as *clearance* was determined. This parameter represents the solute removal rate and is defined as follows:

(39) $$Clearance=\frac{\left(Q_{Bin}\times C_{Bin}-Q_{Bout}\times C_{Bout}\right)}{C_{Bin}}$$

With the data obtained for each simulated case, a linear regression of the clearance parameter was performed as a function of radial viscous resistance (Equation (40)) using Excel software.

(40) $$Clearance=a\times ln\left(\frac{1}{\alpha_{y}}\right)+b$$

where a and b are constants to be determined.

After determining the parameters a and b of Equation (40), and using the Clearance obtained experimentally by Liao et al. [18], a value of $1/\alpha_{y}$ was obtained and implemented in Ansys Fluent^®^ software. With these data, a new simulation was made, where a new Clearance was obtained, which was compared with the one obtained experimentally by Liao et al. [18] under the same operating conditions. Once the error of this comparison was verified, the value of $1/\alpha_{y}$ was corrected and the process was repeated until a minimum error was obtained (trial and error method).

## 3. Results and Discussion

### 3.1. Mesh Quality Assessment

**Figure 5:**
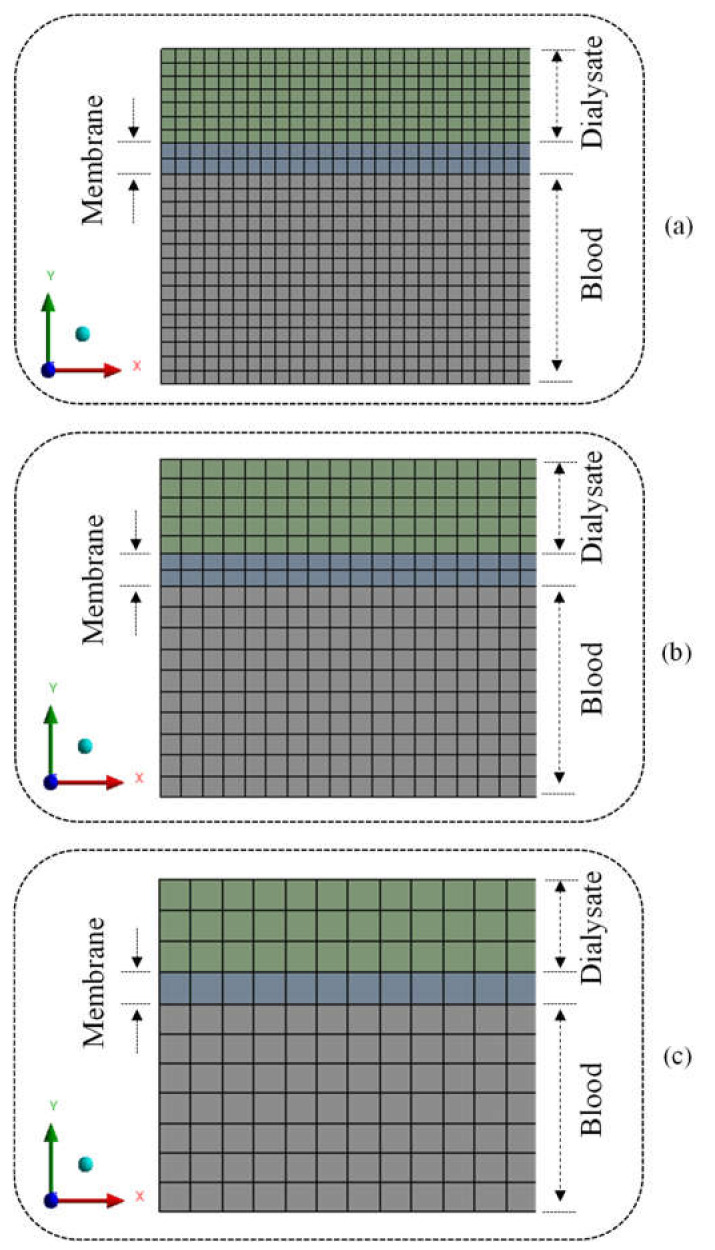
Details of the meshes used in the simulations (**a**) $M_{1}$, (**b**) $M_{2}$, and (**c**) $M_{3}$.

As already mentioned, the Grid Convergence Index (GCI) method was used to evaluate the quality of the developed meshes. To perform the analyses, three computational meshes with different refinement levels were generated ($M_{1}$, $M_{2}$, $M_{3}$) under a refinement ratio of 1.5, applied to the Sizing command in the mesh generation software, Meshing by Ansys^®^. This refinement ratio is per the methodology proposed by Roache [26]. The number of elements of the meshes used can be seen in Table 3. The meshes were made in a structured way, with a standardized refinement throughout the domain; details can be seen in Figure 3 and Figure 5.

**Figure 6:**
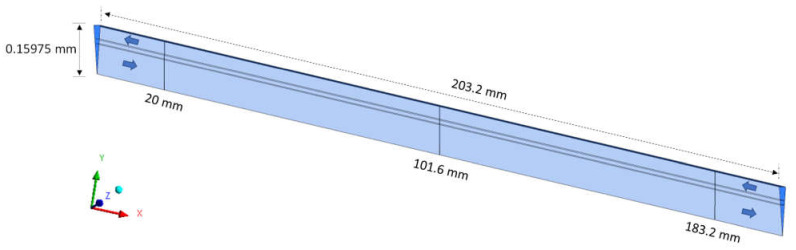
Axial positions of lines for collecting urea velocity data.

For the mesh test, the hydrodynamic variables of urea velocity and pressure were investigated using the GCI. For the analysis of urea velocity using the GCI method, three radial lines were chosen at the axial positions 20.0 mm, 101.6 mm, and 183.2 mm, as shown in Figure 6, aiming to analyze the regions close to the inlet and outlet and center of the equipment.

Table 6 presents the results obtained in the study of the GCI for the urea velocity in the *y* = 0.159 mm position, at the axial positions *x* = 20 mm, 101.6 mm, and 183.2 mm. After analyzing Table 6, it is observed that the values of the coefficient c are in the range of 0<c<1, indicating monotonic convergence. Furthermore, the $GCI_{21}<GCI_{32}$ indicates that the dependence of the results on the mesh elements sizes has been reduced and is approaching an independence condition. It can also be seen that the values of $GCI_{21}$ and $GCI_{32}$ are below the 10% limit, as established by Celik and Karatekin [27]. The values of the variables $GCI_{32}$ and $r_{p}GCI_{21}$ are close enough, indicating that the extrapolated solution is close enough to the exact solution for this variable.

It is observed that, as the mesh is refined, the value of the variable of interest approaches the value of the asymptotic solution. This behavior is evidenced in the three axial positions. Meshes $M_{2}$ and $M_{1}$ present similar solutions and a slight difference is verified for mesh $M_{3}$, with less refinement, especially in the axial position 101.6 mm.

**Figure 7:**
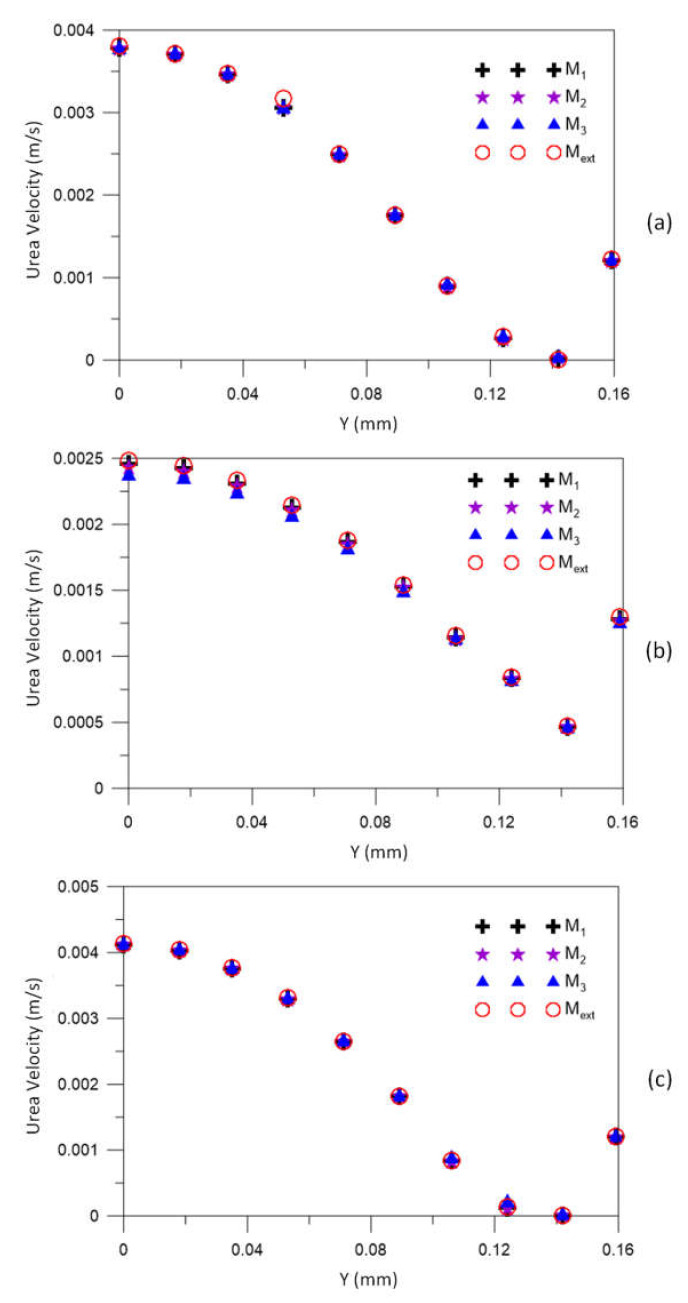
The urea velocity profile in (**a**) 20.0 mm, (**b**) 106.6 mm, and (**c**) 183.2 mm axial positions for different meshes.

Figure 7 illustrates the urea velocity profiles in the three axial positions: 20.0 mm, 101.6 mm, and 183.2 mm, for different meshes $M_{1}$, $M_{2}$, $M_{3}$, compared to the result obtained for the extrapolated mesh ($M_{ext}$). Very similar velocity profiles can be seen in the three figures. For all meshes, velocity values close to the asymptotic solution (obtained with the extrapolated mesh) are verified. Furthermore, it is possible to observe a decrease in the urea velocity with the *Y* position, especially in the range 0.100≤Y≤0.115 mm, due to the resistance to flow, imposed by the porous medium (membrane).

Table 7 summarizes the obtained mean relative error of the variable compared to the obtained value using the extrapolated mesh, for the three axial positions $x_{1}$, $x_{2}$, and $x_{3}$. There is a smaller mean relative error for the most refined mesh ($M_{1}$) compared to the extrapolated mesh, with values of 1.44%, 0.82%, and 0.2%. Similar results are observed for the mesh $M_{2}$, with mean relative errors close to those obtained for $M_{1}$.

**Figure 8:**
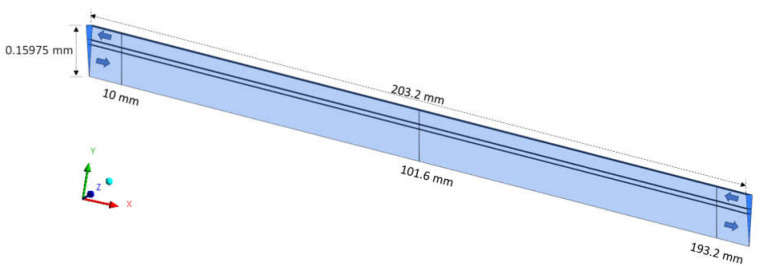
Axial positions of lines for collecting pressure data.

To ensure the quality (convergence) of the analyzed meshes and assist in decision making, in addition to urea velocity, one more hydrodynamic variable was evaluated: the pressure. Thus, the pressure was investigated using the GCI method, in the axial positions 10.0 mm, 101.6 mm, and 193.2 mm, as shown in Figure 8.

Table 8 shows the pressure values at positions $x_{1}$, $x_{2}$, and $x_{3}$, as well as the parameters used in the GCI method. It is observed that the parameter c is in the range between 0 and 1, indicating monotonic convergence, with a solution within the asymptotic range, as expected for $GCI_{32}≅r_{p}GCI_{21}$ [26]. Parameters $GCI_{21}$ and $GCI_{32}$ have values below 10%, satisfying the specifications for convergent solutions as proposed by Celik and Karatekin [27]. Furthermore, the $GCI_{21}$ is smaller than the $GCI_{32}$, indicating independence of the results related to the refinement of the mesh. Analogous behavior can be observed for the other two axial positions, $x_{2}$ and $x_{3}$.

It is observed that meshes, $M_{1}$, $M_{2}$ and $M_{3}$ have similar pressure values, especially meshes $M_{1}$ and $M_{2}$, with a pressure difference of 2.83 Pa at position $x_{1}$, 0.93 Pa at position $x_{2}$, and 1.37 Pa at position $x_{3}$. These behaviors indicate that the solutions obtained with the analyzed meshes are close to the extrapolated solution.

**Figure 9:**
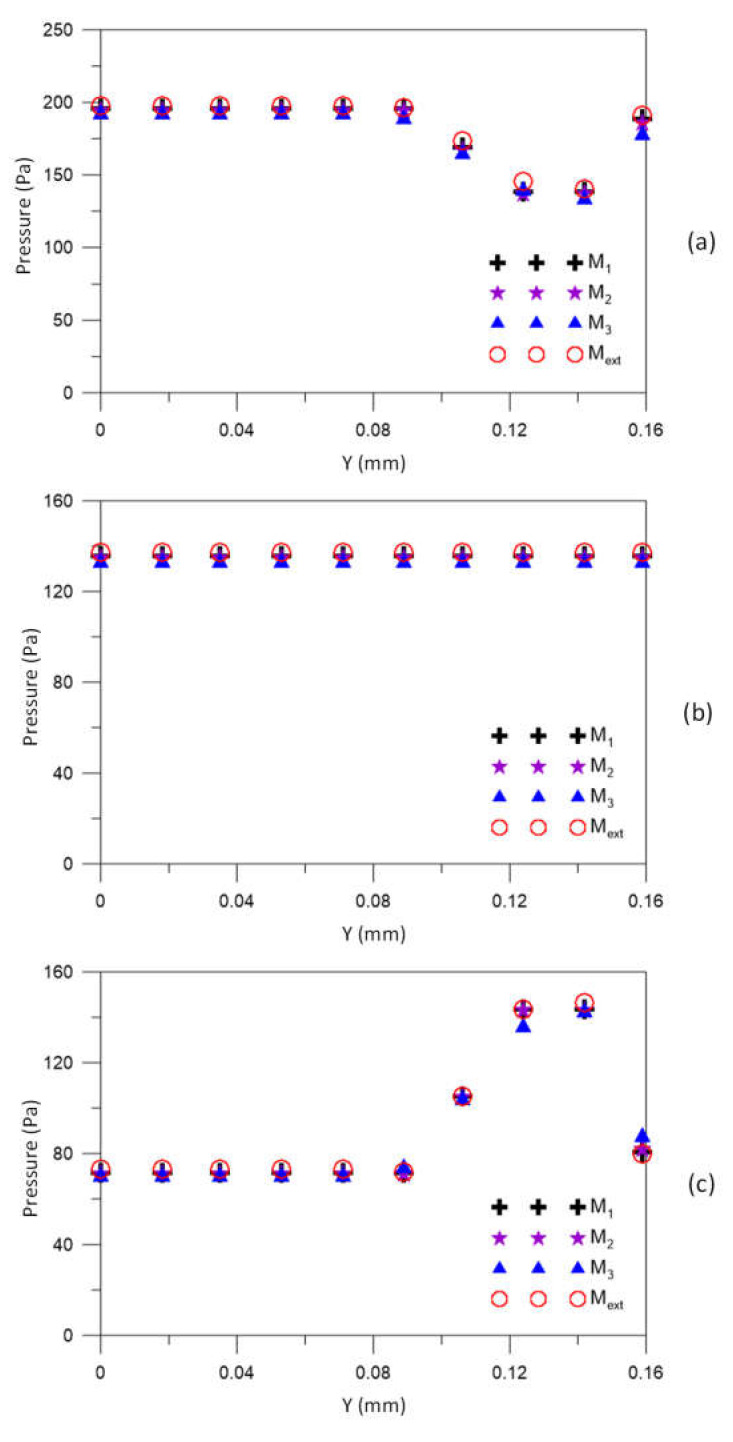
Pressure profile at (**a**) 10.0 mm, (**b**) 106.6 mm, and (**c**) 193.2 mm axial positions for different meshes.

Figure 9 illustrates the pressure profiles at three axial positions $x_{1}$ = 10.0 mm, $x_{2}$ = 101.6 mm and $x_{3}$ = 193.2 mm, at position *y* = 0.159 m, for different mesh sizes $M_{1}$, $M_{2}$, $M_{3}$, compared to the result obtained for the extrapolated mesh, $M_{ext}$. These figures show a good approximation of the solutions obtained with the meshes $M_{1}$, $M_{2}$, and $M_{3}$ with those obtained with asymptotic solutions ($M_{ext}$). Since the fluids are flowing in countercurrent, there are higher pressure values in the regions close to the lumen and shell inlets.

Table 9 illustrates the mean relative error compared to that obtained with the extrapolated mesh, for the three axial positions: 10.0 mm, 101.6 mm, and 193.2 mm, for meshes $M_{1}$, $M_{2}$, and $M_{3}$. Corroborating the results presented in Figure 9 it is observed that the mesh $M_{1}$ has the lowest mean relative error compared to the extrapolated mesh, with values lower than 2%, at the three positions analyzed. A similar result is observed for the mesh $M_{2}$, which presents mean relative errors close to the values obtained for $M_{1}$. However, the mesh $M_{2}$ presents a lower computational effort, with reduced simulation time compared to that observed for the mesh $M_{1}$, in addition to solutions independent of the element size. It is, therefore, the best option for subsequent simulations.

### 3.2. Hollow Fiber Membrane Analysis

#### 3.2.1. Clearance

As a representative parameter of the volumetric flux of urea removed from the bloodstream, clearance was used to validate the mathematical model developed. The statistical parameters of Equation (40) achieved after adjustment to the clearance data obtained in the simulation of cases 04 to 08 are a = −10.19 mL/min and b = 559.54 mL/min. The coefficient of determination obtained after fitting was R^2^ = 0.84.

**Figure 10:**
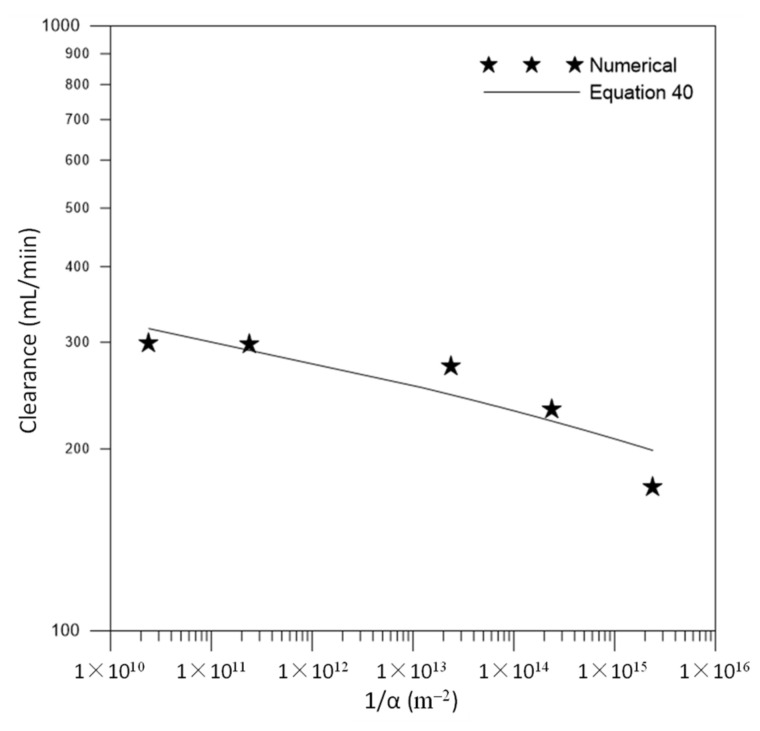
Clearance as a function of membrane viscous resistance.

Figure 10 illustrates the behavior of clearance as a function of the radial viscous resistance of the membrane obtained in cases 04 to 08. It is observed that the clearance decreases with increasing viscous resistance since the permeation through the membrane included in the mathematical model is controlled by the action of the viscous resistance, responsible for regulating the transport of particles in the porous domain. Therefore, when viscous resistance is increased, there is a decrease in the flow through the membrane and, consequently, a reduction in the removal rate of urea from the bloodstream.

Table 10 summarizes the comparison between the best result obtained for clearance after the adjustment process applying the trial and error technique (Case 9) and the experimental and numerical data reported by Liao et al. [18], which were obtained under the same operating conditions.

Upon analyzing Table 10, it is verified that the clearance obtained after successive simulations (Case 9) presents a value close to the experimental result reported by Liao et al. [18], with a very small error, 0.08%, much lower than the error obtained by Liao et al. [18], 6.81%. This difference is probably due to the difference in the mathematical models used in this research and the one used by Liao et al. [18]. In their research, Liao et al. [18] use a methodology based on the coupling of the domains (shell, lumen, and membrane), considering the shell domain as a porous zone, with the Darcy equations being applied to predict the flux on the shell side, the Navier–Stokes equations to simulate lumen-side flux, and the Kedem–Katchalsky equations to calculate transmembrane flux. The present work, on the other hand, uses a multiphase Eulerian–Eulerian approach for all domains, incorporating an extra resistance in the linear momentum equation to predict the flow in the porous domain, making it, therefore, a more robust and accurate model.

#### 3.2.2. Volume Fraction

**Figure 11:**
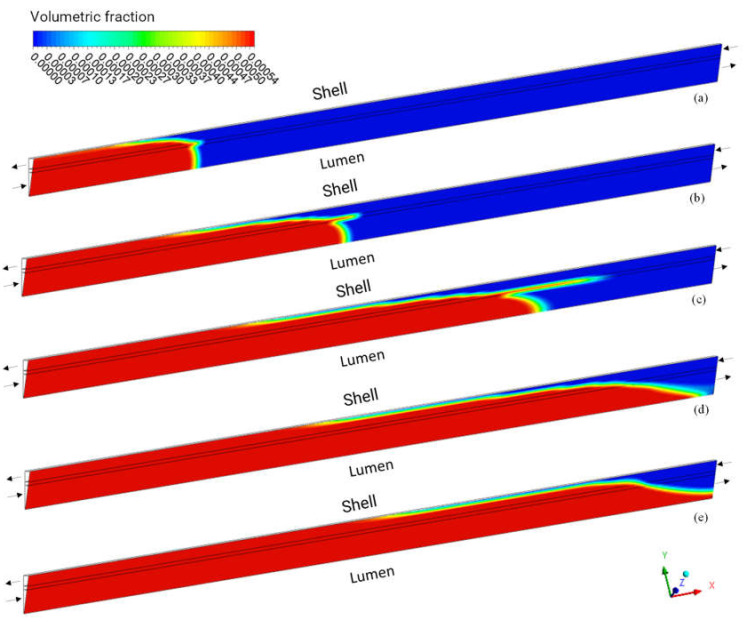
Distribution of the volume fraction of urea in the XY plane at Z = 0 m and different process times: (**a**) 500 s, (**b**) 1000 s, (**c**) 1500 s, (**d**) 2000 s, and (**e**) 2500 s.

Figure 11 shows the urea volume fraction field in the XY plane at Z = 0, for different moments of the process—500, 1000, 1500, 2000, and 2500 s—for case 09. Analyzing this figure, it is observed that the fluid takes approximately 2500 s to travel through the entire hollow fiber membrane, with higher concentrations of urea, as predicted, in the vicinity of the lumen inlet region. In the membrane, it is possible to see the axial permeation of urea due to the characteristic of the anisotropic porous medium, with a lower axial viscous resistance than the radial one (Figure 11a–c); however, this behavior is not maintained for t > 1500 s, due to the approximation of the flow to the shell inlet.

**Figure 12:**
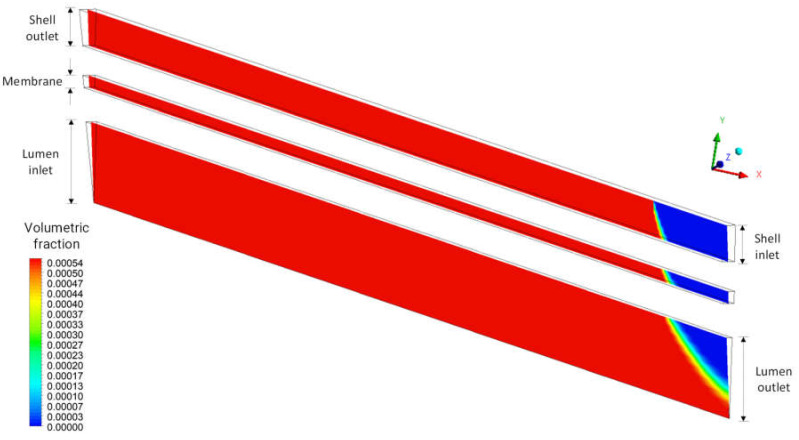
Distribution of the volume fraction of urea in the XY plane at Z = 0 m and at time t = 6200 s.

Figure 12 shows the urea volume fraction field in the domains: shell, porous membrane, and lumen, in the XY plane at Z = 0, at time t = 6200 s, for case 09. It is observed a decrease in the volume fraction of urea at the end of the blood flow region and the beginning of the dialysate flow region. This behavior is associated with the countercurrent flow and the characteristics of the porous medium, which provide permeation of the solute, with higher concentrations of urea in the feed and dialysate output.

**Figure 13:**
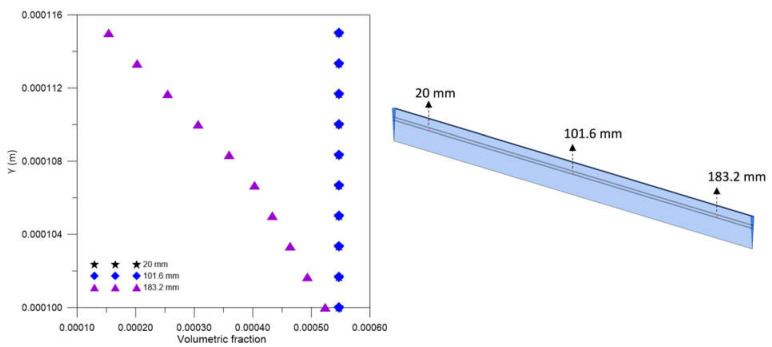
Volume fraction profile of urea inside the membrane, in the axial positions of 20 mm, 101.6 mm, and 183.2 mm, at t = 6200 s (Case 9).

Figure 13 shows the urea volume fraction profile in three axial positions—20.0 mm, 101.6 mm, and 183.2 mm—inside the porous domain, at t = 6200 s. A practically constant volume fraction profile is observed in the axial positions of 20.0 mm and 101.6 mm, and a decreasing behavior with the radial position only in the position of 183.2 mm, precisely due to the proximity to the dialysate inlet in countercurrent.

**Figure 14:**
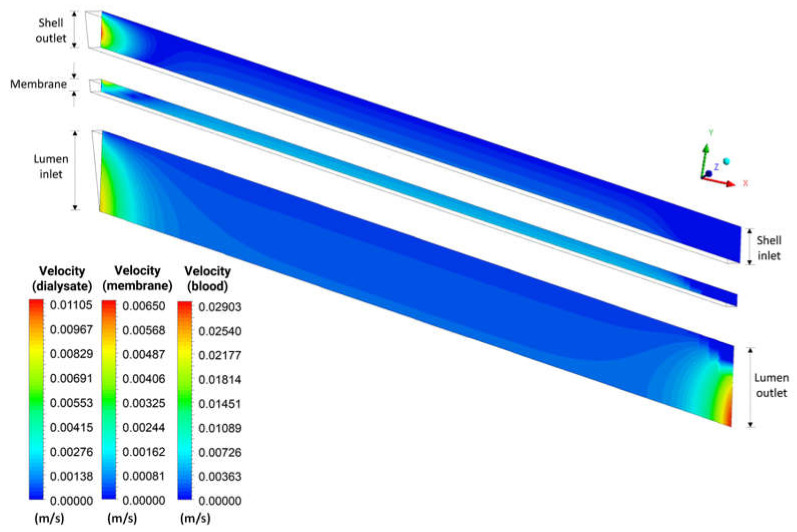
Distribution of the local velocity of urea in the XY plane at Z = 0 m and at time t = 6200 s.

Figure 14 illustrates the urea local velocity field in the domains: shell, lumen, and porous membrane, in the XY plane at Z = 0, at time 6200 s, for case 9. Upon analyzing this figure, higher local urea velocities are observed in the inlet and outlet sections of the lumen, and the exit region of the shell. Countercurrent fluxes, through a membrane with low resistance to flow and the drag force, help transport the solute, causing larger volume fractions of urea in high-velocity regions. Furthermore, variations in velocity inside the porous medium can be seen, associated with the anisotropic membrane, with axial viscous resistance ($7.75\times 10^{8}\;m^{-2}$) greater than the radial viscous resistance ($2.15\times 10^{14}\;m^{-2}$), providing greater permeation along the membrane. It is important to note that the scale of values in the legend is different.

**Figure 15:**
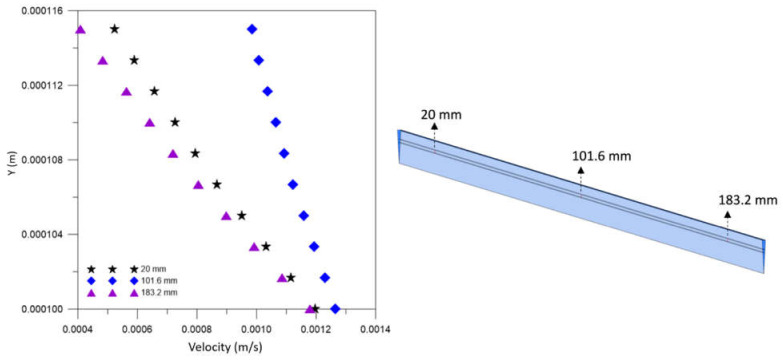
Velocity profile of urea inside the membrane, in the axial positions 20.0 mm, 101.6 mm, and 183.2 mm, at t = 6200 s (Case 9).

Figure 15 shows the urea velocity profile in three axial positions—20.0 mm, 101.6 mm, and 183.2 mm—inside the porous domain at t = 6200 s. Analyzing this figure, we can observe that the urea velocity decreases with the increase of radial position, due to the resistance imposed on the flow by the porous membrane. In addition, there is a similar behavior in the three positions analyzed, with a greater velocity gradient in the axial position 183.2 mm.

**Figure 16:**
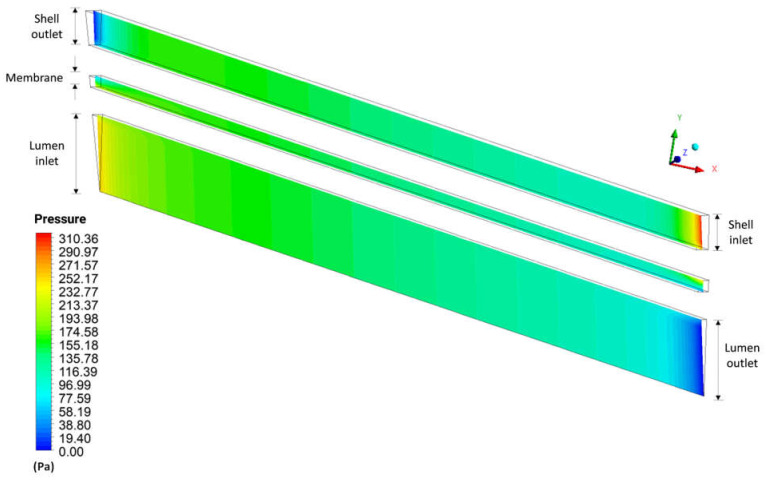
Pressure distribution in the XY plane at Z = 0 m and at time t = 6200 s.

Figure 16 represents the pressure field inside the domains of shell, lumen, and porous membrane, in the XY plane at Z = 0, at time 6200 s, for case 9. Analyzing this figure, high pressures are verified in the entrance regions of the domains, due to the mass flow in these regions, presenting a greater pressure variation in the shell region. This pressure distribution inside the equipment is associated with strict control of the dialyzer output conditions. High inlet pressures help in convective transport of solute, especially in the region close to the lumen inlet, as well as in the appearance of a possible retrofiltration in the vicinity of the lumen outlet. 

**Figure 17:**
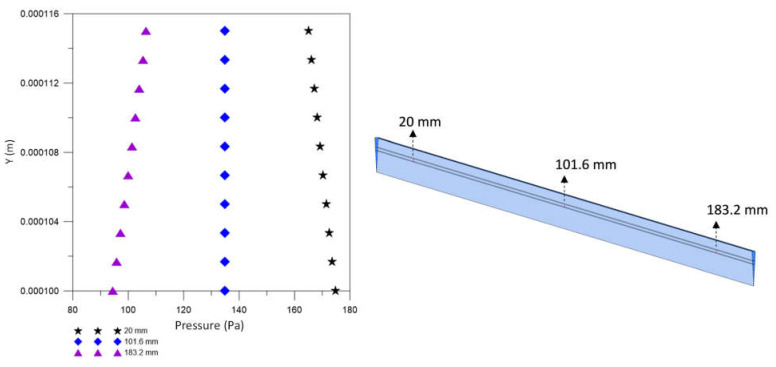
Pressure profile inside the membrane, in the axial positions of 20.0 mm, 101.6 mm, and 183.2 mm, at t = 6200 s (Case 9).

Figure 17 shows the pressure profile in three axial positions, 20.0 mm, 101.6 mm, and 183.2 mm, inside the porous domain, at t = 6200 s. It is observed in the axial position 20.0 mm that the pressure decreases with the increase in the radial position, opposite behavior to that verified in the position 183.2 mm. In these regions, pressure gradients are accentuated due to the influence of equipment inputs and outputs, as seen in the pressure fields (Figure 16). Note that this opposite pressure behavior at positions 20.0 and 183.2 mm causes fluid flow in opposite directions, from the lumen to the shell at x = 20.0 mm and from the shell to the lumen at x = 183.2 mm.

#### 3.2.3. Flow Lines and Velocity Vectors

**Figure 18:**
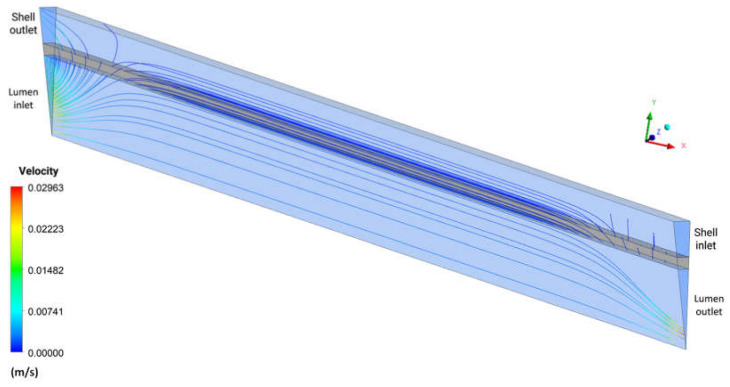
Urea streamlines in the XY plane, at Z = 0 m, and at time t = 6200 s.

Figure 18 shows the behavior of urea velocity in the form of streamlines in the domains: shell, lumen, and porous membrane, in the XY plane at Z = 0, at time 6200 s, for case 9. It is observed, by the streamlines, a behavior similar to that of Figure 14, with the greater mass flow in the region close to the lumen inlet. Furthermore, it is possible to visualize the drag of particles and solute permeation through the membrane, in addition to the presence of the retrofiltration phenomenon.

**Figure 19:**
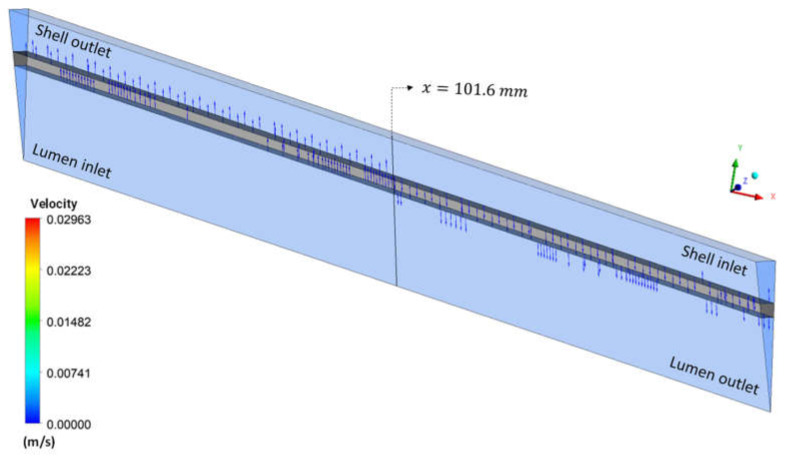
Urea velocity vectors at the hollow fiber membrane interface at t = 6200 s.

Figure 19 shows the urea velocity vectors field at the fiber-hollow membrane interface in the Y direction, for case 9. It is possible to observe a higher urea permeation in the first half of the membrane, reducing with the progress of the flow due to the influence of countercurrent dialysate flow. The retrofiltration phenomenon is evident when observing the velocity field in the form of vectors; it can be seen that the fluid flows from the lumen to the shell in the left region of the domain and the opposite occurs in the right region of the domain. This behavior is expected for hemodialysis membranes, as observed by Eloot [28].

## 4. Conclusions

Based on the numerical results obtained in the simulations of the hemodialysis process via hollow fiber membrane, it can be concluded that the proposed mathematical model proved to be useful to predict the fluid behavior inside the hollow fiber membrane, allowing a better understanding of the fluid dynamics inside the equipment. The clearance parameter decreases with increasing radial viscous resistance and the analysis of the volume fraction field and streamlines of urea showed greater permeation in the vicinity of the lumen inlet region. Moreover, the highest pressures were observed at the shell and lumen inlets, with a greater variation on the shell side, due to the presence of the hollow fiber membrane separating the two domains. Further, the presence of retrofiltration phenomena was observed, especially in the vicinity of the lumen outlet region, due to the influence of countercurrent dialysate feeding.

## Figures and Tables

**Table 1:** Dimensions of the hollow fiber membrane [18].

Equipment Dimensions (mm)	
Length (L)	203.2
Section thickness (E)	0.0208962
The thickness of the dialysate flow region $\left(E_{d}\right)$	0.04475
Membrane thickness $\left(E_{m}\right)$	0.015
Blood flow thickness $\left(E_{b}\right)$	0.1

**Table 2:** Thermo-physical properties and parameters of fluids and membrane [18].

Fluids		Density ρ (kg/m^3^)	Viscosity μ (kg/m·s)	Viscous Resistance Axial $1/\alpha_{x}$ (m ^−2^)	Porosity
Dialysate		998.2	0.001003	-	-
Blood	Water	998.2	0.001003	-	-
	Urea	1280.0	0.002300	-	-
Membrane		-	-	$7.75\times 10^{8}$	0.2

**Table 3:** Simulated cases for the GCI analysis.

Case	Number of Mesh Elements $\left(N_{m}\right)$
01	718.920
02	344.267
03	147.785

**Table 4:** Parameters considered constant in the GCI analysis.

Parameter	Symbol	Value
Lumen feed flux (mL/min)	$Q_{Bin}$	300
Shell feed flux (mL/min)	$Q_{Din}$	300
Axial viscous resistance (m^−2^)	$1/\alpha_{x}$	$7.75\times 10^{8}$
Radial viscous resistance (m^−2^)	$1/\alpha_{y}$	$2.15\times 10^{14}$
Urea concentration in the lumen feed (kg/m^3^)	$C_{in}$	0.7

**Table 5:** Conditions used in hollow fiber membrane simulations.

Case	$1/\alpha_{y}\;\left(m^{-2}\right)$
04	$2.40\times 10^{10}$
05	$2.40\times 10^{11}$
06	$2.40\times 10^{13}$
07	$2.40\times 10^{14}$
08	$2.40\times 10^{15}$
09	$2.15\times 10^{14}$

**Table 6:** Parameters obtained from the study of the Grid Convergence Index for urea velocity as response variable (*y* = 0.159 m).

Parameter		Axial Position		
		$x_{1}$ = 20 mm	$x_{2}$ = 101.6 mm	$x_{3}$ = 183.2 mm
Urea velocity (m/s)	Mesh *M_1_*	$3.709\times 10^{-3}$	$2.422\times 10^{-3}$	$4.036\times 10^{-3}$
	Mesh *M_2_*	$3.706\times 10^{-3}$	$2.399\times 10^{-3}$	$4.034\times 10^{-3}$
	Mesh *M_3_*	$3.698\times 10^{-3}$	$2.340\times 10^{-3}$	$4.030\times 10^{-3}$
p		1.384	1.509	1.831
$\phi_{ext}^{21}=M_{ext}$ (m/s)		$3.712\times 10^{-3}$	$2.411\times 10^{-3}$	$4.037\times 10^{-3}$
$ICM_{21}$		$1.067\times 10^{-3}$	$1.031\times 10^{-2}$	$2.778\times 10^{-4}$
$ICM_{32}$		$2.104\times 10^{-3}$	$2.179\times 10^{-2}$	$6.815\times 10^{-4}$
c		0.394	0.368	0.308
$r_{p}ICM_{21}$		$2.102\times 10^{-3}$	$2.159\times 10^{-2}$	$6.813\times 10^{-4}$

**Table 7:** Relative error compared to the extrapolated mesh.

Mesh	Mean Relative Error (%)		
	$x_{1}$ (20 mm)	$x_{2}$ (101.6 mm)	$x_{3}$ (183.2 mm)
$M_{1}$	1.44	0.82	0.2
$M_{2}$	2.04	1.66	0.36
$M_{3}$	1.50	3.86	0.59

**Table 8:** Parameters obtained from the study of the Grid Convergence Index for pressure as response variable (*y* = 0.159 m).

Parameter		Axial Position		
		$x_{1}$ = 10.0 mm	$x_{2}$ = 101.6 mm	$x_{3}$ = 193.2 mm
Pressure (Pa)	Mesh *M_1_*	188.42	135.71	80.58
	Mesh *M_2_*	185.58	134.78	81.96
	Mesh *M_3_*	177.54	132.85	87.39
p		1.598	1.030	2.205
$\phi_{ext}^{21}=M_{ext}$ (Pa)		190.81	137.14	79.88
$ICM_{21}$		$1.58\times 10^{-2}$	$1.31\times 10^{-2}$	$1.09\times 10^{-2}$
$ICM_{32}$		$3.52\times 10^{-2}$	$2.19\times 10^{-2}$	$3.16\times 10^{-2}$
c		0.350	0.483	0.252
$r_{p}ICM_{21}$		$3.47\times 10^{-2}$	$2.17\times 10^{-2}$	$3.22\times 10^{-2}$

**Table 9:** Mean relative error compared to extrapolated mesh.

Mesh	Mean Relative Error (%)		
	$x_{1}$ (10.0 mm)	$x_{2}$ (101.6 mm)	$x_{3}$ (193.2 mm)
*M_1_*	1.46	1.04	1.17
*M_2_*	2.30	1.72	1.56
*M_3_*	4.00	3.13	1.90

**Table 10:** Comparison between results obtained in this work and those available in the literature.

Case	Radial Viscous Resistance, $1/\alpha \left(m^{-2}\right)$	Clearance (mL/min)				
		Numerical (This Work)	Experimental (Liao et al. [16])	Error	Numerical (Liao et al. [18])	Error
09	$2.15\times 10^{14}$	235.18	235±7.4	0.08%	220	6.81%

## Data Availability

The data that support the findings of this study are available upon request from the authors.
